# Acidogenic Potential of Oral *Bifidobacterium* and Its High Fluoride Tolerance

**DOI:** 10.3389/fmicb.2019.01099

**Published:** 2019-05-16

**Authors:** Ayumi Manome, Yuki Abiko, Junko Kawashima, Jumpei Washio, Satoshi Fukumoto, Nobuhiro Takahashi

**Affiliations:** ^1^Division of Pediatric Dentistry, Tohoku University Graduate School of Dentistry, Sendai, Japan; ^2^Division of Oral Ecology and Biochemistry, Tohoku University Graduate School of Dentistry, Sendai, Japan; ^3^Division of Community Oral Health Science, Department of Community Medical Supports, Tohoku Medical Megabank Organization, Tohoku University, Sendai, Japan

**Keywords:** glycolysis, metabolome, children, caries, acetate, *Streptococcus mutans*

## Abstract

*Bifidobacterium* is frequently detected in early childhood caries and white spot lesions, indicating that it is a novel caries-associated bacterium. *Bifidobacterium* is known to possess a unique metabolic pathway, the “bifid shunt,” which might give it cariogenic potential by increasing its acid production. Thus, we evaluated the acid-producing activity of *Bifidobacterium* and its sensitivity to fluoride, a caries preventive reagent. *Bifidobacterium longum*, *Bifidobacterium dentium*, and *Streptococcus mutans* were used. Acid-producing activity was measured using a pH-stat in the absence and presence of fluoride under anaerobic conditions. Furthermore, metabolomic analysis was performed to elucidate the mechanism underlying the inhibitory effects of fluoride. The acid production of *Bifidobacterium* at pH 5.5 was as high as that seen at pH 7.0, indicating that *Bifidobacterium* has high cariogenic potential, although it produced less acid than *S. mutans*. In addition, *Bifidobacterium* produced acid in the absence of extracellular carbohydrates, suggesting that it can store intracellular polysaccharides. *Bifidobacterium* produced more acid from lactose than from glucose. *Bifidobacterium* mainly produced acetate, whereas *S. mutans* mainly produced lactate. The 50% inhibitory concentration (IC_50_) of fluoride for acid production was 6.0–14.2 times higher in *Bifidobacterium* than in *S. mutans*. Fluoride inhibited enolase in the glycolysis, resulting in the intracellular accumulation of 3-phosphoenolpyruvate, glucose 6-phosphate, and erythrose 4-phosphate. However, the bifid shunt provides a bypass pathway that can be used to produce acetate, suggesting that *Bifidobacterium* is able to metabolize carbohydrates in the presence of fluoride. It is suggested that its exclusive acetate production contributes to the pathogenesis of dental caries.

## Introduction

The Bifidobacteriaceae family consists of 7 genera: *Aeriscardovia, Alloscardovia, Bifidobacterium*, *Gardnerella, Metascardovia*, *Parascardovia*, and *Scardovia*, which have been isolated from both animals and humans. These bacteria are Gram-positive anaerobic bacilli, which in humans are mainly found in the gastrointestinal tract (Scardovi, 1986). They are known to have beneficial effects, such as reducing the number of harmful bacteria via the host and enhancing intestinal immunity (Gibson and Wang, 1994; Gill et al., 2001).

In recent years, the development of *Bifidobacterium*-selective medium has revealed that it is also present in the oral cavity (Beighton et al., 2008). Ten *Bifidobacterium* species have been isolated from the oral biofilm (Modesto et al., 2006) and two of these species, *Bifidobacterium longum* and *Bifidobacterium dentium*, have also been isolated from saliva (Beighton et al., 2008). It was reported that *Bifidobacterium* was detected in 80% of the plaque samples from early childhood caries patients (Aas et al., 2008; Tanner et al., 2011). In addition, *Bifidobacterium* was detected in the saliva of children, and its frequency was higher in children with caries (95%) than in caries-free children (9%) (Kaur et al., 2013). Based on these facts, oral *Bifidobacterium* has been recognized as a novel caries-associated bacterium. The predominant *Bifidobacterium* species in the oral cavity is *B. dentium* (Munson et al., 2004). *B. longum* is also frequently detected in the human oral cavity (Nyvad and Kilian, 1990; Aas et al., 2008; Mantzourani et al., 2009).

Furthermore, our laboratory reported that these bacterial species have a series of abilities that make them highly acid-resistant. For example, they exhibit a higher survival rate and are more able to maintain a constant intracellular pH than *Streptococcus mutans* and *Lactobacillus paracasei* (Nakajo et al., 2010). Among the various acid resistance mechanisms employed by bacteria, the ability to produce acids under acidic conditions is one of the important caries-inducing factors, and most of the acids produced under such conditions are derived from carbohydrate metabolism. *Bifidobacterium* species are known to possess a unique metabolic pathway, the “bifid shunt” (Ruas-Madiedo et al., 2005; Sánchez et al., 2005). Most dental caries-related bacteria, such as *S. mutans*, metabolize carbohydrates through glycolysis (the Embden-Meyerhof-Parnas pathway) and mainly produce lactate (Takahashi, 2015), whereas *Bifidobacterium* species were reported to metabolize carbohydrates through the bifid shunt, which produces both acetate and lactate as end-products (Lee and O’Sullivan, 2010). However, it is still unclear how caries-related *B. longum* and *B. dentium* metabolize carbohydrates and produce acids under physiological conditions in the oral cavity.

In addition, fluoride is widely used to prevent caries in various ways around the world. It has been reported that fluoride inhibits enolase, which is one of the glycolytic enzymes used by most oral bacteria, such as oral *Streptococcus*, *Actinomyces*, and *Lactobacillus* (Kashket et al., 1977; Hamilton and Ellwood, 1978; Marsh et al., 1985; Hata et al., 1990; Maehara et al., 2005), leading to the subsequent repression of bacterial acid production and growth. While enolase is encoded and expressed in the bile-resistant derivative strain of *B. longum* (Sánchez et al., 2005), its fluoride sensitivity is unknown.

Thus, in this study we focused on the carbohydrate metabolism of novel caries-associated bacteria, *B. longum* and *B. dentium*, and examined their acid-producing activity and the suppression of carbohydrate metabolism by fluoride (including the biochemical mechanism responsible for its suppression), while considering the physiological conditions of the oral cavity.

## Materials and Methods

### Bacterial Strains and Growth Conditions

*Bifidobacterium dentium* JCM 1195 and *B. longum* subsp. *longum* JCM 1217 were provided by the RIKEN BRC through the National Bio-Resource Project of the MEXT, Japan. *B. dentium*, *B. longum*, and *S. mutans* NCTC 10449 were used in this study. These bacteria were maintained on CDC anaerobe blood agar (Nippon BD, Tokyo, Japan) at 37°C in an anaerobic glove box (N_2_, 80%; H_2_, 10%; CO_2_, 10%; NHC-Type; Hirasawa Works, Tokyo, Japan). The bacteria were cultured in a complex medium containing 1.7% tryptone (Becton Dickinson, Franklin Lakes, NJ, United States), 0.3% yeast extract (Becton Dickinson), 0.5% NaCl, 50 mM phosphate buffer solution (PPB, pH 7.0), 0.5% glucose (TYG medium), or 0.5% lactose (TYL medium) at 37°C in an NHC-type glove box, as described previously (Nakajo et al., 2010). The bacteria grown in TYG were used for the experiments examining glucose metabolism, while TYL was used in the experiments investigating lactose metabolism.

The bacterial cells were harvested by centrifugation at the logarithmic growth phase [optical density (OD) at 660 nm: 0.8–0.9] (*Bifidobacterium*: 15,000 × *g*, 10 min, 4°C; *Streptococcus*: 21,000 × *g*, 7 min, 4°C) using double-sealed centrifuge tubes to maintain anaerobic conditions. Then, they were washed with 2 mM PPB (pH 7.0) containing 150 mM KCl and 5 mM MgCl_2_, before being resuspended in the same buffer at an OD at 660 nm of 3.5 and stored at 4°C until use. The washing and preservation of the cells were carried out under anaerobic conditions in an anaerobic glove box (N_2_, 90%; H_2_, 10%; NH-Type; Hirasawa Works).

### Bacterial Acid Production and the Effects of Fluoride

The reaction mixture was set to a pH-stat (AUTO pH-stat; model AUT-211S, Toa Electronics, Tokyo, Japan) and pre-incubated at 37°C for 15 min at pH 7.0 or 5.5. At pH 5.5, the pH was adjusted by adding 0.12 N HCl to the reaction mixture, as described previously (Kawashima et al., 2013). Potassium fluoride was added to the reaction mixture at a final concentration of 0–80 mM (1 mM = 19.1 ppm F). After further pre-incubation for 5 min, acid production was started by adding glucose or lactose at a final concentration of 10 mM. The rate of acid production was monitored for 10 min based on a titration volume of 60 mM KOH using the pH-stat. All of these experiments were performed under anaerobic conditions (in an NH-type anaerobic box). Moreover, the 50% inhibitory concentration (IC_50_) of fluoride was calculated. The IC_50_ was determined by the following calculation from the data presented in Figure 3. IC_50_ = 10ˆ[LOG(A/B)^∗^(50-C)/(D-C)+LOG(B)], where A is a higher concentration across 50%, B is a lower concentration across 50%, C is an inhibition rate (%) at B and D is an inhibition rate (%) at A.

### Acidic End-Products

As mentioned above, the acid production was measured by pH-stat. Subsequently 0.45 mL of the reaction mixture (OD at 660 nm: 3.5) was sampled and immediately mixed with 0.05 mL of 6 N perchloric acid to terminate bacterial carbohydrate metabolism. The samples were then removed from the anaerobic box and filtered through a polypropylene membrane (pore size: 0.20 μm; Toyo Roshi Ltd., Tokyo, Japan). The filtrates were quantitatively analyzed by high performance liquid chromatography (HPLC; Shimadzu Prominence LC-20AD, Shimadzu Co., Ltd., Kyoto, Japan). The concentrations of the acidic end-products lactate, formate, and acetate were quantitatively analyzed, as described previously (Takahashi et al., 1987; Norimatsu et al., 2015).

### Metabolome Profile

The *B. dentium* suspension was incubated with glucose for 2 min, as described above, in the presence or absence of 20 mM (pH 7.0) or 2 mM (pH 5.5) potassium fluoride. To clearly determine inhibitory steps by fluoride, 20 mM and 2 mM, a fluoride concentration at which acid production was inhibited by about 50%. Before and after it was incubated, the reaction mixture was sampled and immediately centrifuged at 10,000 rpm for 2 min, before the cell fraction and supernatant were separated. The metabolites in the cells were extracted from the cell fraction and pre-treated, as reported previously (Takahashi et al., 2010), before being analyzed using capillary electrophoresis time-of-flight mass spectrometry (CE-TOFMS; G1600AX and G1969A; Agilent Technologies, Waldbronn, Germany). A fused silica capillary column (H3305-2002; Human Metabolome Technologies, Tsuruoka, Japan) was used to separate the metabolites during the CE. The analysis was performed in negative ion mode. The separated metabolites were then mixed with sheath liquid (H3302-1020; Human Metabolome Technologies) and continuously sent to the TOFMS system for mass analysis (Soga et al., 2002, 2003). The obtained metabolomic data were analyzed using specific software (MassHunter workstation, Agilent Technologies, CA, United States). The following metabolites were targeted: glucose 6-phosphate, fructose 6-phosphate, 3-phosphoglycerate, phosphoenolpyruvate, and pyruvate for glycolysis, and erythrose 4-phosphate, sedoheptulose 7-phosphate, ribose 5-phosphate, and ribulose 5-phosphate for the bifid shunt.

### Enzyme Assay and Its Inhibition by Fluoride

*Bifidobacterium dentium* and *S. mutans* were harvested by centrifugation at the logarithmic growth phase. The pellet was washed twice with 2 mM PPB (pH 7.0) containing 150 mM KCl and 5 mM MgCl_2_ and stored at −80°C until used. After being thawed, the cells were suspended in 2 mM PPB containing 1 mM dithiothreitol and disrupted by sonic oscillation for 10 min at 4°C (200 W, 2 A; Insonator, Kubota, Japan). Cell debris was removed by centrifugation (10,000 × *g*, 10 min, 4°C) and resultant cell-free extracts (CFE) were assayed for enzyme reaction.

The assay mixture for glucose phosphate isomerase (EC 5.3.1.9) contained 5 mM fructose 6-phosphate, 0.7 U/ml glucose 6-phosphate dehydrogenase, 10 mM NADP and CFE in 2 mM PPB (pH 7.0) (Walter et al., 1983). The assay mixture for transaldolase (EC 2.2.1.2) contained 1 mM erythrose 4-phosphate, 10 mM fructose 6-phosphate, 0.4 mM NADH, 1.5 U/ml glycerophosphate dehydrogenase, 1.2 kU/ml triosephosphate isomerase, 8 mM EDTA, 5 mM MgCl_2_ and CFE in 80 mM triethanolamine buffer (pH 7.0) (Walter et al., 1983). The assay mixture for enolase (EC 4.2.1.11) contained 20 mM 2-phosphoglycerate and CFE in 2 mM PPB (pH 7.0) (Guha-Chowdhury et al., 1997; Takahashi et al., 1997). Enzyme activity of enolase was measured by following the change in absorbance of phosphoenolpyruvate photometrically at 240 nm using a spectrophotometer (UV-1800, Shimadzu Co., Ltd., Kyoto, Japan). Similarly, the enzyme activity of glucose phosphate isomerase and transaldolase was measured by the change in absorbance of NADP at 340 nm. Inhibition experiments were conducted with 0, 0.02, 0.2, and 2 mM potassium fluoride.

### Statistical Analyses

The data are expressed as mean and standard deviation values and were analyzed using the paired *t*-test, the paired *t*-test combined with Bonferroni’s correction, or Dunn’s test. Differences associated with *P*-values of < 0.05 were considered to be statistically significant.

## Results

### Acid Production by *Bifidobacterium*

**FIGURE 1:**
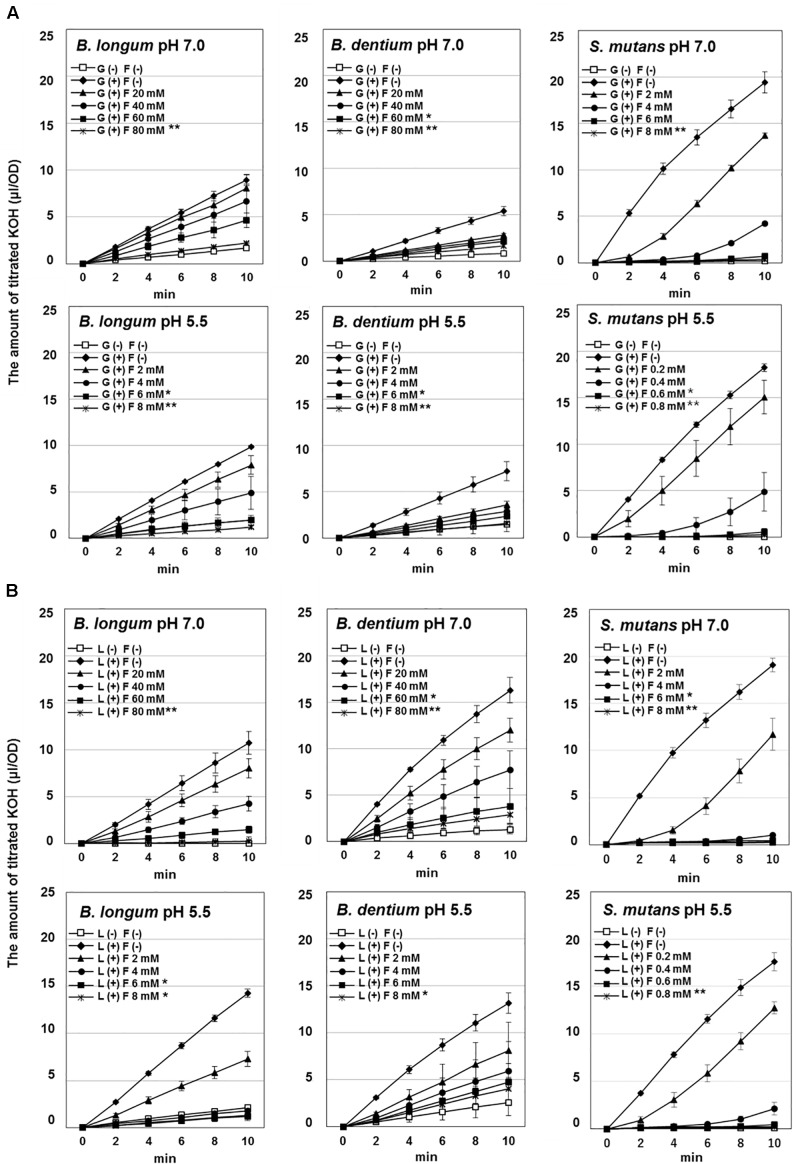
Effect of fluoride on the acid production by *Bifidobacterium longum*, *Bifidobacterium dentium*, and *Streptococcus mutans* at pH 7.0 and 5.5. **(A)** Acid production from glucose. **(B)** Acid production from lactose. The data are shown as the mean and standard deviation of three independent experiments. Significant differences in the amount of acid production in the presence of fluoride were analyzed by comparison with that in the absence of fluoride at 10 min (Dunn test; ^∗∗^*P* < 0.01, ^∗^*P* < 0.05).

**FIGURE 2:**
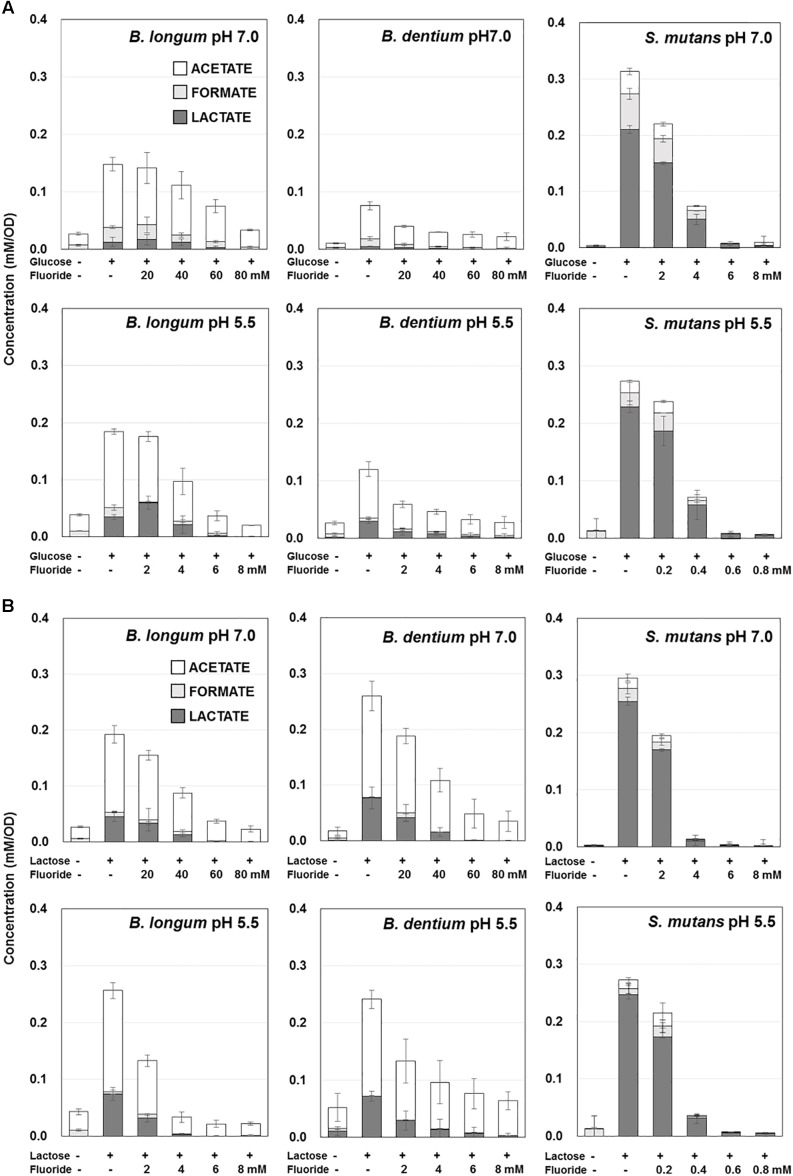
Effect of fluoride on the acidic end-products by *B. longum*, *B. dentium*, and *S. mutans* at pH 7.0 and 5.5. **(A)** Acidic end-products from glucose. **(B)** Acidic end-products from lactose. The data are shown as the mean and standard deviation of three independent experiments. The concentration of acidic end-products was the value per OD of the cell suspension (OD = 3.5).

The acid production of each bacterial strain from glucose or lactose was monitored based on the titration volume of alkali (KOH) solution using a pH-stat (Figure 1), and the acidic end-products that accumulated in the reaction mixture were analyzed and quantified by HPLC (Figure 2). The amounts of acid detected by the pH-stat corresponded to the total amount of acidic end-products, indicating that the acidic end-products were comprehensively detected and precisely quantified.

The amounts of acid produced from glucose or lactose by *Bifidobacterium* were higher at pH 5.5 than at pH 7.0 (Figure 1, 2). On the other hand, the amounts of acid produced by *S. mutans* were lower at pH 5.5 than at pH 7.0. In addition, *Bifidobacterium* produced a larger amount of acid from lactose than from glucose, whereas *S. mutans* produced similar amounts of acid from glucose and lactose. Furthermore, *S. mutans* exhibited greater acid production from both glucose and lactose than *Bifidobacterium*. However, *Bifidobacterium* demonstrated low level, but continuous acid production in the absence of extracellular carbohydrates.

*Bifidobacterium* mainly produced acetate from glucose, whereas *S. mutans* mainly produced lactate (Figure 2A). In addition, the proportion of lactate increased at pH 5.5 in both *Bifidobacterium* and *S. mutans*. A similar tendency was observed for lactose metabolism. However, for *Bifidobacterium* the proportion of lactate was higher in the presence of lactose than in the presence of glucose (Figure 2B).

### Effects of Fluoride on the Acid Production of *Bifidobacterium*

**FIGURE 3:**
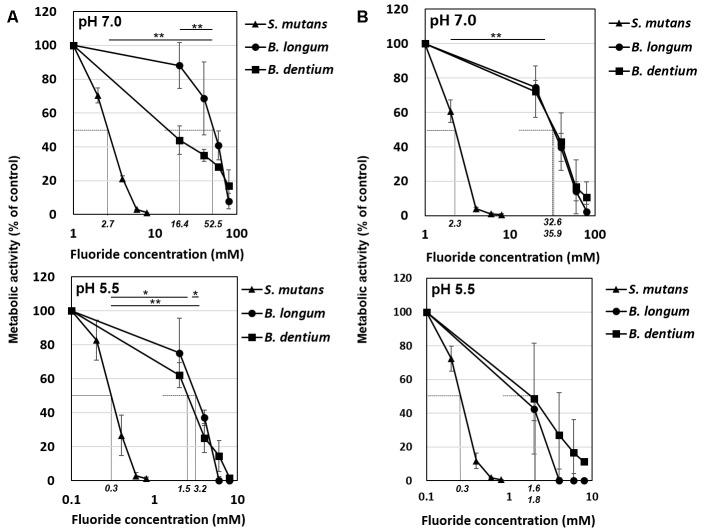
Inhibitory effects of fluoride on bacterial acid production at pH 7.0 and 5.5. **(A)** Acid production from glucose. **(B)** Acid production from lactose. Fifty percent inhibitory concentrations (IC_50_) were calculated from the fluoride concentration vs. relative acid production curve. Data are shown as the mean and standard deviation of three independent experiments. The IC_50_ values are displayed in italics on the *x*-axis. The significance of differences in IC_50_ among *B. longum*, *B. dentium*, and *S. mutans* were analyzed using the Tukey test (^∗∗^*P* < 0.01, ^∗^*P* < 0.05).

The acid production of both *Bifidobacterium* and *S. mutans* decreased in a fluoride concentration-dependent manner (Figure 2). The 50% inhibitory concentration (IC_50_) of fluoride was obtained from the fluoride concentration vs. relative acid production curve (Figure 3). The IC_50_ for *Bifidobacterium* acid production was 6.0–14.2 times higher than that for *S. mutans* acid production, regardless of the pH or metabolic substrate. The proportions of acidic end-products remained almost constant in the presence/absence of fluoride (Figure 2).

### Effects of Fluoride on the Metabolome Profile of *B. dentium*

**FIGURE 4:**
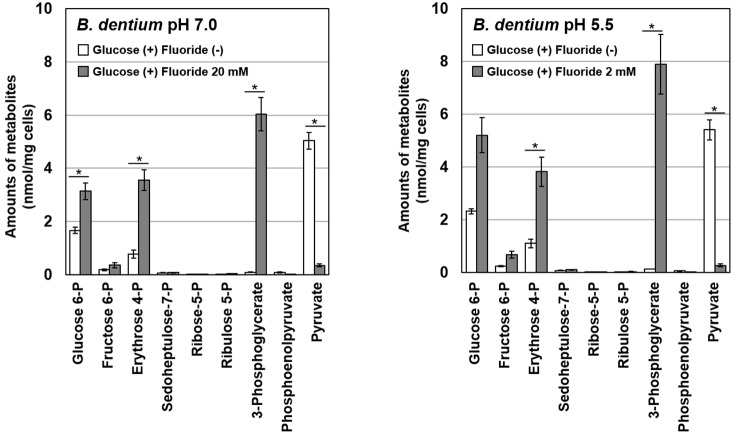
Effects of fluoride on the metabolome profile of *B. dentium* at pH 7.0 and 5.5. Data are shown as the mean and standard deviation of three independent experiments. The significance of differences between with and without fluoride conditions were analyzed using the paired *t*-test. The level of significance was set at 0.0125 based on Bonferroni’s correction (^∗^*P* < 0.0125).

To identify the particular steps in metabolic pathways that were inhibited by fluoride, metabolomic analysis of glucose metabolism in *B. dentium* in the presence/absence of fluoride was performed using CE-TOFMS. After the addition of fluoride, the intracellular accumulation of glucose 6-phosphate and erythrose 4-phosphate in the bifid shunt and 3-phosphoglycerate in glycolysis was observed (Figure 4). On the contrary, the intracellular level of pyruvate was significantly decreased in the presence of fluoride.

### Effects of Fluoride on Enzyme Activity

All the enzymes, enolase, phosphoglucose isomerase and transaldolase were detected; however, only enolase was inhibited by fluoride (data not shown). There was no difference in the sensitivity to fluoride of enolase between *B. dentium* and *S. mutans* and 2 mM potassium fluoride inhibited enolase activity completely in both bacterial strains (Table 1).

**Table 1:** Inhibition of enolase activity by fluoride.

Fluoride concentration	Inhibition of enolase activity by fluoride (%)	
(mM)	*B. dentium*	*S. mutans*
0.02	10.4 ± 2.5	7.6 ± 3.3
0.2	49.4 ± 4.7	53.2 ± 2.8
2	96.2 ± 1.0	94.9 ± 2.5

*Data are shown as the mean ± standard deviation of three independent experiments.*

## Discussion

The acid production of *Bifidobacterium* at pH 5.5 was as high as that seen at pH 7.0, indicating that *Bifidobacterium* has high cariogenic potential, although its acid production was lower than that of *S. mutans* (Figure 1, 2). In addition, *Bifidobacterium* was able to produce acid in the absence of extracellular carbohydrates (Figure 1, 2), suggesting that it can store intracellular polysaccharides and degrade them into acids (producing energy) under carbohydrate-limited conditions, such as between meals, although further and intensive experiments are needed to confirm. On the contrary, *S. mutans* did not produce acid in the absence of extracellular carbohydrates (Figure 1, 2), indicating that *S. mutans* cannot accumulate intracellular polysaccharides under the growth conditions employed in the present study (grown with 0.5% glucose or lactose and harvested at the logarithmic growth phase). *Streptococcus* is known to only accumulate intracellular polysaccharides in the presence of a relatively higher concentration of extracellular carbohydrates after its growth enters the late-logarithmic to stationary phase of growth (Takahashi et al., 1991). *Bifidobacterium* stored intracellular polysaccharides at a relatively low concentration of extracellular carbohydrates at the logarithmic growth phase, potentially contributing to its cariogenic potential.

It is reported that the acid production was measured by pH-drop after the biofilm of *S. mutans* UA159 was formed with the addition of sucrose. As a result, the half maximum effective concentration (EC_50_) of sodium fluoride was 6.85 ppmF (Pandit et al., 2013). While in the present study, the IC_50_ of *S. mutans* was 2.7 mM ( = 52 ppmF) at pH 7.0 and 0.3 mM ( = 5.7 ppmF) at pH 5.5 (Figure 3). In the pH-drop study, it is suggested that the inhibition by the fluoride gradually increased as the pH was lowered. Although simple comparisons cannot be made due to differences in experimental conditions, it is suggested that the acid production could be suppressed at low fluoride concentration as shown in the present study.

**FIGURE 5:**
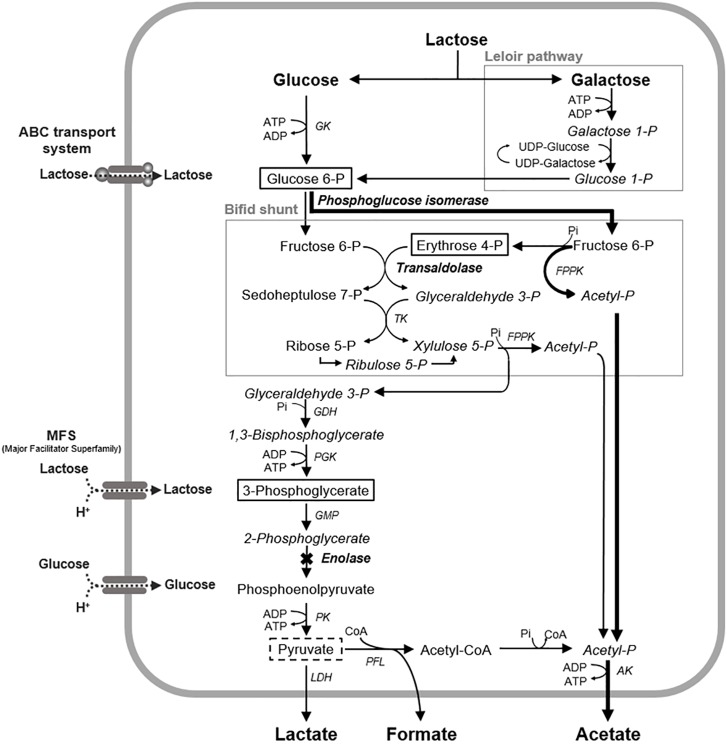
Proposed mechanism of the fluoride inhibition of glucose metabolism in *B. dentium*. The levels of the metabolites framed by solid lines increased in the presence of fluoride, and the levels of the metabolites framed by broken lines decreased in the presence of fluoride. X mark, a metabolic step inhibited by fluoride; Bold arrowed lines, a proposed bypass pathway for acetate production; AK, acetate kinase; GDH, glyceraldehyde 3-phosphate dehydrogenase; GK, glucokinase; PGM, phosphoglycerate mutase; LDH, lactate dehydrogenase; PGK, phosphoglycerate kinase; PFL, pyruvate formate-lyase; PK, pyruvate kinase; TK, transketolase; FPPK, fructose 6-phosphate phosphoketolase; CoA, coenzyme A.

*Bifidobacterium* exhibited greater acid production from lactose than from glucose (Figure 1, 2). *Bifidobacterium* is known to incorporate lactose via the ATP transport system and to degrade it into glucose and galactose (Lee and O’Sullivan, 2010). Galactose is further metabolized to glucose 6-phosphate and enters the bifid shunt via the Leloir pathway (Figure 5; Lee and O’Sullivan, 2010). It is reported that some species of *Bifidobacterium* preferentially utilizes lactose when glucose and lactose are both available (Parche et al., 2006), and *Bifidobacterium* demonstrated slightly higher growth in lactose than in glucose (Krzewinski et al., 1996). Lactose uptake and the subsequent Leloir pathway might be more efficient than the corresponding glucose pathway, although a detailed study is needed to confirm this. In addition, the fact that *Bifidobacterium* is able to efficiently produce acids from lactose might explain why it is often detected in the oral cavities of children (Aas et al., 2008; Mantzourani et al., 2009; Tanner et al., 2011) because infants often consume lactose-rich foods, such as breast milk.

It was reported that the acetate:lactate production ratio of *Bifidobacterium* is 3:2 (Lee and O’Sullivan, 2010). However, the present study revealed that *Bifidobacterium* produced much more acetate than lactate (at ratios of 2:1–4:1) and also produced formate (Figure 2). This finding can probably be explained by the fact that the current study was conducted under strict anaerobic conditions simulating the oral biofilm environment, where pyruvate formate lyase (PFL), an oxygen-sensitive enzyme, is maintained in an active state and produces formate (Figure 5; Abbe et al., 1991). PFL can also produce acetyl-CoA, which subsequently leads to acetate production via acetyl phosphate (Figure 5). The present study is the first to show that *Bifidobacterium* produces formate and has an exclusive acetate production pathway.

Acetic acid is a weaker acid than lactic acid and has a higher acid dissociation constant (*p*Ka = 4.8) than lactate (*p*Ka = 3.8) so it is more non-ionized than lactic acid in low pH environments; i.e., the dissociation equilibrium equation of acetic acid shifts further toward the left (AH ⇄ A^−^ + H^+^). The non-ionized form of an acid (AH) is more likely to penetrate into dental enamel than the ionized form (A^−^), and once an acid penetrates into enamel, a hydrogen ion is released, causing demineralization within the enamel (Hoppenbrouwers and Driessens, 1988). This suggests that *Bifidobacterium*, which is an acetate-producing bacterium, might induce and promote dental caries through a different mechanism from lactate-producing bacteria, such as *S. mutans* and *Lactobacillus* species.

In acetate production via the bifid shunt and the PFL pathway, ATP is generated from acetyl phosphate (Figure 5). The bifid shunt is reported to provide 2.5 molecules of ATP from each molecule of glucose (Lee and O’Sullivan, 2010), which is more productive than the lactate pathway involving glycolysis (which generates 2 ATP molecules per glucose molecule). The PFL pathway supplies one additional molecule of ATP via pyruvate (Abbe et al., 1982; Figure 5). The efficiency of these energy-generating pathways suggests that *Bifidobacterium* can be competitive under carbohydrate-limited conditions in the oral cavity.

The IC_50_ of fluoride for *Bifidobacterium* acid production was much higher than that for *S. mutans* acid production, regardless of the substrate provided and the pH (Figure 3). Metabolomic analysis revealed the significant accumulation of 3-phosphoglycerate, glucose 6-phosphate, and erythrose 4-phosphate in the presence of fluoride (Figure 4), suggesting that the metabolic steps catalyzed by enolase and phosphoglucose isomerase in glycolysis and transaldolase in the bifid shunt were inhibited by fluoride, but the inhibition was weaker than that of *S. mutans* (Figure 5). However, the enzyme assay revealed that fluoride inhibited only enolase and that there was no difference in the fluoride sensitivity between *B. dentium* and *S. mutans* (Table 1). These results indicate that the reductions in the amounts of lactate and formate seen in the presence of fluoride in both *B. dentium* and *S. mutans* (Figure 2) was due to the fact that fluoride inhibits enolase and decreases the supply of pyruvate, a precursor of lactate and formate (Figure 5). In *Bifidobacterium*, the inhibition of enolase can also induce the accumulation of intracellular metabolites in the upstream pathways including the bifid shunt, especially glucose 6-phosphate and erythrose 4-phosphate which intrinsically tend to accumulate (see the metabolic profile in the absence of fluoride in Figure 4). However, there is a bypass pathway from fructose 6-phosphate to acetate via acetyl phosphate in the bifid shunt (Figure 5, bold lines), so *Bifidobacterium* is able to escape the fluoride-induced metabolic inhibition and continue to produce acetate and ATP.

## Conclusion

The present study revealed that *Bifidobacterium* exhibits aciduric and fluoride-tolerant acid production. The high fluoride tolerance of *Bifidobacterium* was thought to be due to its unique metabolic pathway, the bifid shunt, which can provide a bypass pathway. Its exclusive acetate production pathway suggests that weaker acids other than lactate might be involved in the etiology of caries. In addition, the efficient lactose-based acid production seen in *Bifidobacterium* might explain why this bacterium is often detected in the oral cavities of children, who often consume lactose-rich foods, such as breast milk.

## Author Contributions

AM contributed to data acquisition and analysis, drafted and critically revised the manuscript. YA contributed to conception, design, data acquisition, analysis, and data interpretation, drafted and critically revised the manuscript. JK contributed to conception, and data interpretation, for pH-stat experiments and acidic end-product analysis. JW contributed to conception, design, and data interpretation, drafted and critically revised the manuscript for metabolome analysis CE-TOFMS. SF contributed to conception and design the manuscript. NT contributed to conception, design, data interpretation, drafted and critically revised the manuscript. All authors gave final approval and agreed to be accountable for all aspects of the work.

## Conflict of Interest Statement

The authors declare that the research was conducted in the absence of any commercial or financial relationships that could be construed as a potential conflict of interest.
